# TLR Signals in Epithelial Cells in the Nasal Cavity and Paranasal Sinuses

**DOI:** 10.3389/falgy.2021.780425

**Published:** 2021-11-22

**Authors:** Masanobu Suzuki, Clare Cooksley, Takayoshi Suzuki, Mahnaz Ramezanpour, Akira Nakazono, Yuji Nakamaru, Akihiro Homma, Sarah Vreugde

**Affiliations:** ^1^Department of Otolaryngology-Head and Neck Surgery, Faculty of Medicine and Graduate School of Medicine, Hokkaido University, Sapporo, Japan; ^2^Department of Surgery–Otorhinolaryngology Head and Neck Surgery, Central Adelaide Local Health Network and the University of Adelaide, Adelaide, SA, Australia

**Keywords:** chronic rhinosinusitis, allergic rhinitis, NFκB, Poly(I:C), nasal epithelial cells, intracellular zinc, nasal polyps

## Abstract

The respiratory tract is constantly at risk of invasion by microorganisms such as bacteria, viruses, and fungi. In particular, the mucosal epithelium of the nasal cavity and paranasal sinuses is at the very forefront of the battles between the host and the invading pathogens. Recent studies have revealed that the epithelium not only constitutes a physical barrier but also takes an essential role in the activation of the immune system. One of the mechanisms equipped in the epithelium to fight against microorganisms is the Toll-like receptor (TLR) response. TLRs recognize common structural components of microorganisms and activate the innate immune system, resulting in the production of a plethora of cytokines and chemokines in the response against microbes. As the epithelia-derived cytokines are deeply involved in the pathogenesis of inflammatory conditions in the nasal cavity and paranasal sinuses, such as chronic rhinosinusitis (CRS) and allergic rhinitis (AR), the molecules involved in the TLR response may be utilized as therapeutic targets for these diseases. There are several differences in the TLR response between nasal and bronchial epithelial cells, and knowledge of the TLR signals in the upper airway is sparse compared to that in the lower airway. In this review, we provide recent evidence on TLR signaling in the upper airway, focusing on the expression, regulation, and responsiveness of TLRs in human nasal epithelial cells (HNECs). We also discuss how TLRs in the epithelium are involved in the pathogenesis of, and possible therapeutic targeting, for CRS and AR.

## Introduction

The respiratory tract is always at risk of being invaded by microorganisms such as viruses, bacteria and fungi, responsible for various infectious conditions. The airway tract is equipped with several defense mechanisms against these potentially harmful pathogens. These include physical mucosal barriers (consisting of mucin, beating cilia and an epithelial layer) and the innate and adaptive immune responses. Pharyngeal reflexes such as coughing and swallowing also contribute to the removal of invading microorganisms. These mechanisms exclude microbes from the airway, physically, immunologically, and neurophysiologically, to sustain the homeostasis of the respiratory tract. In particular, the immune response takes a pivotal role in preventing microorganisms from entering our body.

To initiate the immune response, pattern recognition receptors (PRRs) act by detecting the invading microorganisms. PRRs recognize structurally conserved molecules derived from microbes and activate downstream signals, resulting in the promotion of expression of many genes. Toll-like receptors (TLRs) are examples of PRRs. There are 10 types of TLRs in humans which share common mechanisms of action. TLRs are expressed in many cell types including monocytes, macrophages, dendritic cells, neutrophils, fibroblasts, endothelial cells, and epithelial cells (1). The expression and responsiveness of each TLR differs depending on the cell type (2). Despite many similarities between the upper and lower airways (the so-called united airway), there are significant differences in the TLR response between nasal and bronchial epithelial cells (2–5). Although the TLR response in the lower airway has been well-documented (1, 6), literature on TLR signaling in the upper airway is relatively sparse.

In this review, we provide recent evidence on TLR signaling in the upper airway, focusing on the expression, regulation, and responsiveness of TLRs in human nasal epithelial cells (HNECs). We also discuss how TLRs in the epithelium are involved in the pathogenesis of, and possible therapeutic targeting, for chronic rhinosinusitis (CRS) and allergic rhinitis (AR).

## Toll-Like Receptors

**Figure 1:**
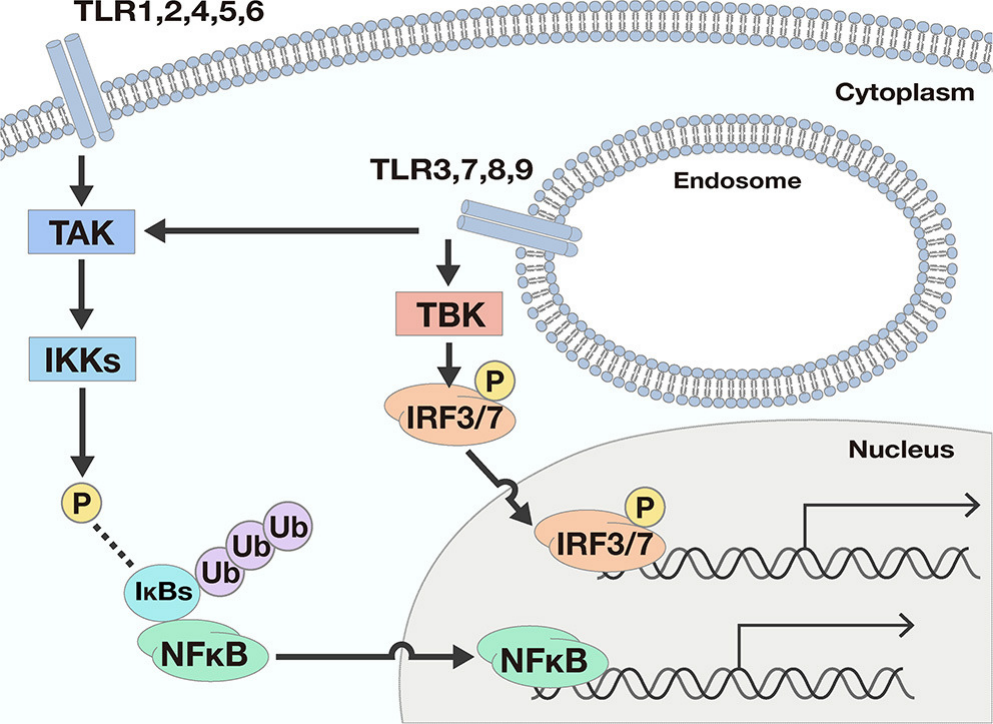
Two downstream signals activated by TLR responses. There are two main downstream signals activated by TLR responses; Nuclear factor-κB (NFκB) and interferon (IFN) signals. NFκB signals are activated by most TLRs. When TLRs recognize ligands, the signal is transferred to activate the IκB kinases (IKKs) by phosphorylation by TGF-β activating kinase (TAK). The activated IKKs phosphorylate IκBs, and the phosphorylated IκBs are degraded by a ubiquitin-proteasome system. Consequently, NFκB is free from IκBs and may translocate into the nucleus, resulting in the promotion of many genes' expression. To the contrary, IFN signals are only promoted by TLR3, 7, 8, and 9. The ligation of TLRs and the corresponding ligand pass through several processes and finally phosphorylate and activate interferon regulatory transcription factor (IRF) 3 or IRF7. Once IRF3 and IRF7 are activated, the transcription factors drive expression from the IFN-β promoter.

TLRs act as a primary sensor for pathogens by recognizing pathogen-derived compounds, which are structurally conserved among (7). Each TLR detects a specific ligand from microorganisms (Table 1). For example, TLR3 explicitly recognizes double-stranded RNA (dsRNA), which is produced by viruses when they reproduce. TLR4 is involved in detecting invading Gram-negative bacteria by recognizing the lipopolysaccharide (LPS) from their outer membrane. TLRs recognize these ligands by their leucine-rich repeats and activate downstream signals through their Toll-IL-1 receptor (TIR) domain (8), resulting in promotion of expression of many genes. Nuclear factor-κB (NFκB) and interferon (IFN) signals are examples of signals induced by TLR responses. NFκB is a transcription factor involved in the promotion of expression of many genes including Interleukin (IL)-6, IL-8, Tumor necrosis factor α (TNFα), and Matrix metalloproteinase 9 (MMP9). The transcriptional activity of NFκB is strictly regulated by the cell, where translocation of NFκB molecules to the nucleus is restricted. In a steady-state, NFκB is captured by inhibitory κB (IκBs) and trapped within the cytosol. When TLRs recognize their respective ligands, the TIR domain and components of the innate signaling pathway transfer the signal to activate two kinases, the IκB kinase α (IKKα) and IKKβ. The activated IKKs phosphorylate IκBs, and the phosphorylated IκBs are degraded by a ubiquitin-proteasome system. Consequently, NFκB is free from IκBs and translocate into the nucleus, resulting in the promotion of many genes' expression (9) (Figure 1).

**Table 1:** Characteristics of TLRs.

	**Ligand**	**Representative Pathogens**	**Localization**	**NFκB Activation**	**IFN Activation**
TLR1	Triacyl lipopeptide	Bacteria	Cell membrane	+	None
TLR2	Diacyl lipopeptide Zymosan	Bacteria Fungi	Cell membrane	+	None
TLR3	dsRNA	Viruses, host cells	Intracellular organelles/cell membrane	+	+
TLR4	Lipopolysaccharide HSP	Bacteria Host cells	Cell membrane	+	None
TLR5	Flagellin	Bacteria	Cell membrane	+	None
TLR6	Diacyl lipopeptide	Mycoplasma	Cell membrane	+	None
TLR7	ssRNA Imidazoquinolines	Viruses Small synthetic compound	Intracellular organelles	+	+
TLR8	ssRNA	Viruses	Intracellular organelles	+	+
TLR9	Unmethylated CpG DNA	Bacteria Viruses	Intracellular organelles	+	+
TLR10	unknown	Unknown	Cell membrane	+	None

*TLR, Toll-like receptor; IFN, interferon; dsRNA, double-strand RNA; HSP, heat shock protein; ssRNA, single-strand RNA*.

As for IFN signals, the ligation of TLRs and the corresponding ligands pass through several processes involving TIR domain-containing adaptor inducing interferon-β (TRIF), TRIF-related adaptor molecule (TRAM), TANK Binding Kinase 1 (TBK), and Myeloid differentiation primary response 88 (Myd88) proteins, and finally activate interferon regulatory transcription factor (IRF) 3 or IRF7. Once IRF3 and IRF7 are activated, expression from the IFN-β promoter is initiated (10).

TLRs can be separated into two groups according to their cellular localization. One group consists of TLR1, 2, 4, 5, and 6, which are responsible for recognition of polysaccharides, lipids, and proteins from microbes. These TLRs activate NFκB signals but not IFN signals. The second group includes TLR3, 7, 8, and 9. These TLRs recognize nucleic acids derived from viruses and activate not only NFκB but also IFN signals (Figure 1).

The two groups have different subcellular localizations. The former group is expressed on cell membranes, while the latter localizes to intracellular organelles such as endoplasmic reticulum and endosomes. One exception is TLR3. Depending on the type of cells, TLR3 is expressed both in intracellular compartments and on the cell surface (11).

TLR expression and responsiveness to TLR ligands varies with cell type (1). In the case of airway epithelial cells, TLR expression in the lower airway has been well-documented (1, 6). Although all TLRs from TLR1 to 10 are expressed in bronchial epithelial cells (12, 13), the most significant activation in those cell types is achieved by TLR3 agonists, Poly (I:C) (12). Besides, lipopolysaccharide (LPS, TLR4 agonist), Zymosan A (TLR2 agonist), peptidoglycans (PGN, TLR2 agonist) and Flagellin (TLR5 agonist) can activate TLRs in airways and promote expression of many genes (12, 14). The molecular expression of TLRs themselves in the lower airway is also induced by TLR ligands. Poly (I:C) induced expression of TLR2, 3, 6, and 10 (12). TLR3 expression is also promoted with TLR4 stimulation by LPS (12). However, the expression of TLRs does not always correlate with their responsiveness to ligands (13, 15).

## TLRs in the Upper Airway

Literature involving upper airway epithelial cells is more sparce than on the lower airway. Several differences in TLR signals have been reported between the upper and lower airway (2–5). Unlike the lower airway, from which cell lines available such as Nuli-1, BEAS-2B, and NCI-H292, no cell lines have been established from the upper airway. Primary cells harvested from the nasal epithelium are often used for research, but these cells have slower growth speed than cell lines. Human nasal epithelial cells (HNECs) only retain their properties up to passage four after which they go into senescence (16). Therefore, the cells are not applicable for experiments involving knockdown, which is one of the basic methods to investigate the molecular mechanisms within cells. These limitations have made the molecular- and cellular- biological research on the upper airway more difficult than the lower airway.

### TLR Expression in HNECs

Several studies have investigated TLR expression in HNECs. Van Tongeren, et al. reported that TLR1-6 and TLR9 were expressed in HNECs but they failed to confirm the expression of TLR7, 8, and 10 (15). Cooksley et al. demonstrated that TLR1, 2, 3, 4, 8, 9, and 10 were expressed in HNECs (2). Tengroth et al. reported TLR3, 7 and 9 expressions in HNECs (17). Despite these apparent discrepancies, these reports indicate that all known TLRs from TLR1 to TLR10 are expressed in HNECs (13). However, this does not imply that all TLR ligands activate innate immune signals in those cells in the same way or to a similar extent. TLR expression can correlate with various TLR-induced cytokines' release (15).

### TLR Signaling in HNECs

So far, several articles have reported the activation of TLR signals in HNECs. Among the TLR ligands, dsRNA, the ligand for TLR3, demonstrates the most significant effect on promotion of gene expression in HNECs, including TNF-α (18), IL-6 (2, 19), IL-8 (18), Thymic stromal lymphopoietin (TSLP) (20), Matrix metalloproteinases (MMPs) and Tissue inhibitor of metalloproteinase (TIMP-1) (21), and Angiotensin-Converting Enzyme 2 (ACE2) and Transmembrane protease, serine 2 (TMPRSS2) (22, 23). The dsRNA-specific-responsiveness in HNECs may be an adaptation of those cells to the frequent exposure to viruses (15, 21). TLR3 specifically recognizes dsRNA, which most viruses synthesize in their life-cycles (24). Considering acute and chronic rhinosinusitis often develop after a viral infection, such as influenza virus, parainfluenza virus, rhinovirus, and respiratory syncytial virus, it would make sense for HNECs lining the nasal cavity to have high sensitivity against dsRNA to activate an early innate immune response against the invading viral infection.

dsRNA is recognized not only by TLR3 but also by Retinoic acid inducible gene-I (RIG-I), another PRR. RIG-I, as well as TLR3, are also expressed in HNECs (17, 21). The length of the dsRNA is thought to determine which receptors recognize it (25, 26). Therefore, different types of viruses are recognized by different PRRs, depending on the length of dsRNA that the virus synthesizes. For example, paramyxoviruses, influenza virus and Japanese encephalitis virus are recognized by RIG-I (27), while rhinovirus and respiratory syncytial viruses activate the innate immune signal through TLR3 (28). However, the contribution of activation through RIG-I of Poly (I:C)-dependent signaling in HNECs may be limited. Indeed, RIG-I-specific agonists, 5'-ppp-dsRNA, failed to promote expression of MMP9, one of the target genes of NFκB. To the contrary, TLR3-specific agonists, polyadenylic acid-polyuridylic acid (Poly A:U), successfully promoted gene expression, similar to Poly(I:C) (21). This result indicates that TLR3, rather than RIG-I, may mediate Poly(I:C) dependent innate immune signaling in HNECs.

In contrast with TLR3-dependent signaling, the role of TLR4 seems limited in HNECs. Although TLR4 is expressed together with key co-stimulatory molecules such as myeloid differentiation factor 2 (MD-2) and CD14 (2, 15), many studies have reported little or no upregulation of gene expression with LPS, a TLR4 agonist (2, 15, 19, 21, 22, 29). Considering that abundant bacteria are localized within the nasal cavity, even at a steady state, the hypo-responsivity to LPS in HNECs might be beneficial to prevent exaggerated inflammation against normal flora in the nasal cavity (15).

Studies show that agonists for TLR1 (22), TLR2 (18, 22), TLR5 (2, 21, 22), TLR6 (2, 21, 22), TLR8 (20), and TLR9 (18, 22, 30) promote signaling in HNECs. However, their influence is relatively limited compared to those that act on TLR3.

As for TLR10, whose ligands have not yet been defined (31), it has not yet been investigated if and how TLR10 regulates downstream pathways and gene expression in HNECs. Different from other TLRs, TLR10 is considered to exhibit anti-inflammatory properties (31). TLR10 reportedly binds to dsRNA and negatively regulates TLR3 signaling by competing with TLR3 for dsRNA ligation (32). Similar suppressive regulation might exist in HNECs.

## Regulators of TLR Signaling in HNECs

To date, several regulators of TLRs and their downstream pathways have been found. These include glucocorticoids (33, 34), epigenetic regulation (21, 35), intracellular zinc levels (36, 37), and the activated TLR signal itself (4, 19).

### Glucocorticoids

**Figure 2:**
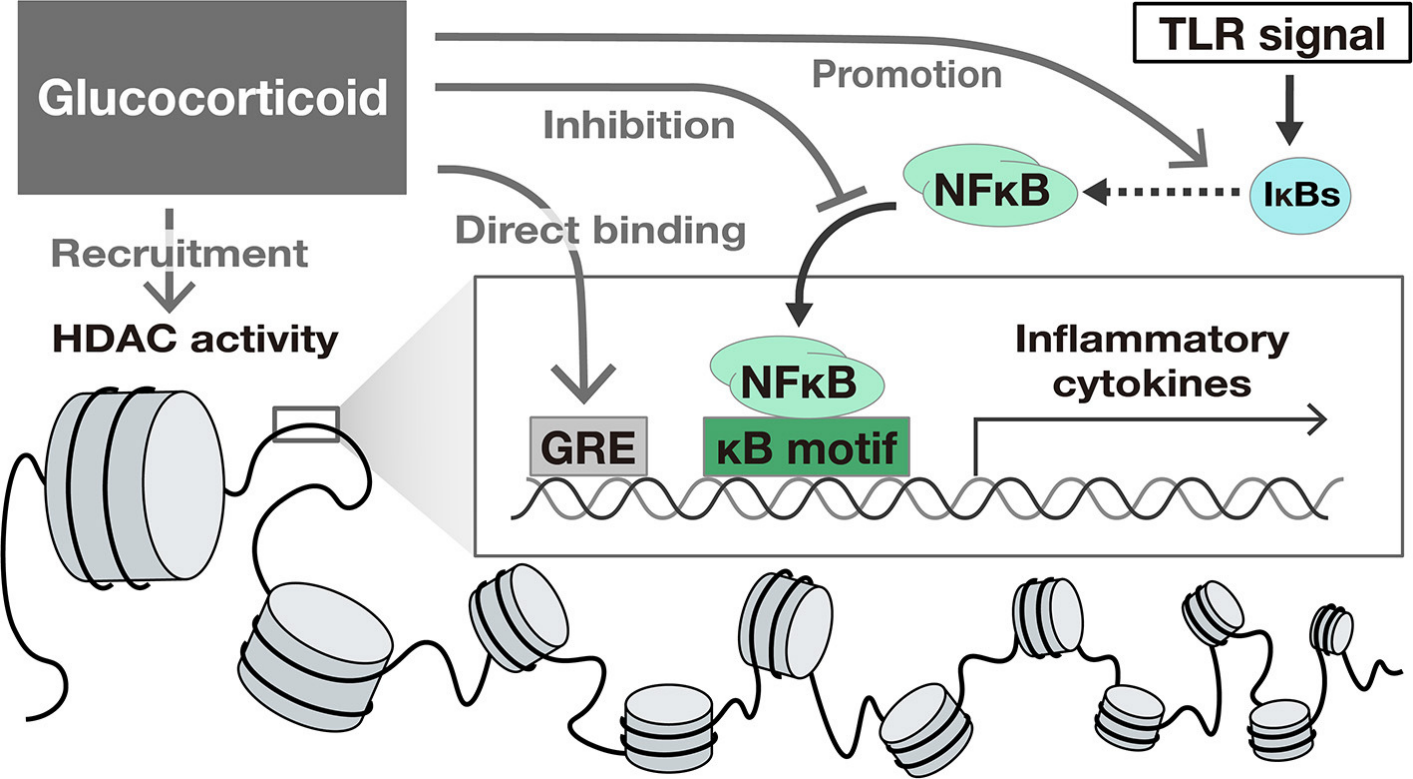
The suppressive effect of Glucocorticoids on TLR-induced gene activation. Glucocorticoids suppress gene transcription by several mechanisms including (1) binding to specific recognition sequences, namely glucocorticoid response elements (GREs) on the DNA, (2) promotion of anti-inflammatory proteins such as IκBs, (3) interference with the translocation of NFκB, and (4) epigenetic modification by recruitment of Histone deacetylases (HDAC) to the transcriptional region.

Glucocorticoids (GC) suppress TLR-induced gene activation. GCs activate glucocorticoid receptors and suppress intracellular signaling pathways such as NFκB and IFN signals, exerting a suppressive effect on the expression of many genes. The mechanisms of the suppression of gene expression by GC include the following: (1) binding to specific recognition sequences, namely glucocorticoid response elements (GREs) on the DNA (38), (2) promotion of anti-inflammatory proteins such as IκBs (39), (3) interference with the binding of other transcriptional factors to promoter regions (33, 34), and (4) epigenetic modification by recruitment of Histone deacetylases (HDAC) (40) to the transcriptional regions (41, 42) (Figure 2).

The regulatory effect of GC on transcription varies depending on the cell type and the downstream pathways (42–44). For example, GC suppressed the expression of IFN-target genes in macrophages, but not in fibroblasts (45). In the case of HNECs, GC suppressed NFκB transcriptional activity and NFκB target genes such as TNF-α and IL-6 (22, 46). In contrast, the significant suppression of IFN-β itself or IFN-stimulated genes by GC have not been identified (22). The suppressive effect of GC on IFN signaling pathway might be limited in HNECs.

Nowadays, glucocorticoids are widely used for the treatment of inflammatory diseases of the nasal cavity and paranasal sinuses such as CRS and AR. Glucocorticoids are applied not only systemically but also locally as intranasal corticoids spray. Especially the intranasal administration is one of the first choices of conservative treatments of CRS and AR due to its confirmed efficacy and safety (47–49).

### Epigenetic Regulation of TLR Signals in HNEC

Gene transcription is also modified by epigenetic modifications, such as DNA methylation and histone modification, which affect the chromatin structure within the nucleus and subsequently gene expression. For example, Sirtuin-1 (SIRT1), a Nicotinamide adenine dinucleotide-dependent deacetylase, modifies core histones in chromatin with its HDAC activity, subsequently winding DNA and resulting in general transcriptional repression (35). In the context of TLR signaling in HNECs, our group reported that SIRT1 suppressed Poly(I:C)-induced MMP9 expression (21). SIRT1 inhibitors significantly increased Poly(I:C)-induced MMP9 expression and activity, while SIRT1 activators decreased them. In addition, MMP9 expression was inversely correlated to SIRT1 expression in nasal mucosa (21). Considering MMP9 expression is regulated by NFκB signals, the suppressive effect of SIRT1 is thought to depend at least in part on the reduction of nuclear translocation and increased deacetylation of NFκB (50–53).

The suppressive effect of MMP9 expression by SIRT1 has also been reported in other types of cells and is considered a common mechanism across different cell types (54–56). Given that SIRT1 is involved in the regulation of the NFκB signaling pathway, apart from MMP9, many NFκB target genes could also be regulated by SIRT1 in HNECs. Indeed, our group found that a SIRT1 activator also suppressed Poly(I:C)-induced IL-6 and TNF-α mRNA (unpublished data). SIRT1 could therefore be a therapeutic target by regulating inflammation and cytokine expression in nasal mucosa, as these target genes are involved in the pathophysiology of inflammatory conditions of the nasal cavity. Interestingly, SIRT1 regulation of MMP9 was not found in the non-inflammatory state, such as HNECs without Poly(I:C) stimulation or nasal mucosa from healthy controls. However, SIRT1 regulation was observed in inflammatory conditions such as HNECs treated with Poly(I:C) or nasal mucosa from CRS (21). This indicated that SIRT1 did not alter the signaling activity in the steady state but did suppress gene expression in inflammatory conditions, which would be preferable for therapeutic application.

### Intracellular Zinc

Another possible factor affecting the TLR signaling is intracellular zinc levels (36, 37). In TLR-induced downstream pathways, signal transduction largely depends on posttranslational modifications of proteins including phosphorylation, dephosphorylation and ubiquitination (57–59). The activity of protein kinases (60), and phosphatases (61–63) is influenced by zinc levels. Furthermore, SIRT1 is dependent on zinc for its molecular structure and, therefore, enzymatic activity (64, 65). Thus, altered zinc homeostasis could influence cytokine production as a result of TLR stimulation (66). Indeed, Poly(I:C)-induced IL-6 and IL-8 were significantly upregulated in HNECs that were incubated in zinc-depleted medium as opposed to those in normal medium (67). This finding supports the notion that impaired zinc homeostasis in HNECs causes enhanced pro-inflammatory cytokine release promoted by activated TLR signals.

In general, intracellular homeostasis of zinc is maintained by zinc transporters (Zinc Iron and Protein; ZIP and Zinc transporter protein; ZnT) and chelators (metallothioneins, MTs). Interestingly, it was recently reported that tissue zinc level is reduced in nasal mucosa from CRS (68). Intracellular zinc depletion was associated with impairment of epithelial barrier structure and function and promoted pro-inflammatory cytokine expression (67, 68). As the vulnerability of epithelial barrier function and the promotion of pro-inflammatory cytokines in nasal mucosa are hallmarks of CRS, impaired zinc homeostasis might be involved in the pathogenesis of CRS (66, 68).

### Other TLR Stimulation

The TLR-induced innate immune signal affects the expression of TLR molecules themselves. In HNECs, Poly(I:C) stimulation increased the expression of TLR1, 2 and 3, while TLR5 expression was decreased (4).

TLR stimulation also alters the responsiveness of TLRs to subsequent signals. Ramezanpour et al. investigated the influence of priming with Poly(I:C) on TLR-agonist-induced IL-6 production. In the experiment, HNEC-Air-liquid Interface (ALI) cultures were primed with Poly(I:C) and after 24 h, the cells were stimulated with Heat Killed *Listeria Monocytogenes* (HKLM), LPS, or Poly(I:C). The results showed that IL-6 was significantly decreased in primed HNECs compared to those without pretreatment (19).

van Tongeren et al. showed the synergistic effect of PGN and Poly(I:C) on IL-6 production in HNECs and bronchial epithelial cells. First, the cells were stimulated with Poly(I:C) and then PGN was added to the cells. Twenty-four h later, the supernatant was collected and subjected to ELISA to quantify IL-6. The IL-6 production in the cells treated with both of Poly(I:C) and PGN was significantly higher than the sum of IL-6 from the cells treated with PGN or cells treated with Poly(I:C) alone (4).

Together, these findings support the notion of crosstalk between TLR3 and various TLRs in HNECs and indicates that activation of TLR3 can affect the expression and potential for activation of not only that same TLR3 over time, but also the responsiveness of other TLRs thereby affecting overall immune activation pathways.

## TLRs in Upper Airway Inflammatory Diseases

TLR signals are deeply involved in inflammatory diseases of the nasal cavity and paranasal sinuses, such as Chronic rhinosinusitis (CRS) and allergic rhinitis.

### Chronic Rhinosinusitis

CRS is a chronic inflammatory condition of the mucosal linings of the nasal cavity and paranasal sinuses. CRS is often divided into two subtypes: chronic rhinosinusitis with nasal polyps (CRSwNP) and without nasal polyps (CRSsNP) (69). Recent evidence showed type 2 inflammatory condition with infiltration of eosinophils is strongly associated with recurrence of nasal polyps after surgery (70, 71). A new classification for CRS has recently been proposed, focusing more on the involvement of the type 2 inflammatory response, dividing CRS into subgroups depending on the presence or absence of type 2 inflammation (47). The type 2 inflammatory response involves induction by type 2 innate lymphoid cells (ILC2) and Th2 cells, which are triggered by several cytokines released from the nasal epithelium as a result of activation of innate immune responses (72). This implicates the nasal epithelium as a critical mediator of immune activation with potential involvement in the pathogenesis of CRS.

#### TLR Expression in CRS

Altered expression of TLRs was reported in nasal mucosa from CRS patients despite some discrepancy between results (73, 74). TLR2 expression was shown to be decreased in both CRSwNP and CRSsNP compared to control (74, 75), although another study showed increased TLR2 expression in both CRSwNP and CRSsNP patients compared to control (76). Interestingly, TLR2 ligand-induced IL-8 promotion was decreased in HNECs from CRS patients compared to those from healthy mucosa (77). Besides TLR2, TLR9 is also well-documented in the inflammatory response in CRS. Decreased TLR9 expression were reported in CRSwNP compared to control (78, 79). The low expression level of TLR2 and TLR9 is also associated with the recurrence of CRSwNP after surgery (75). TLR9 expression in HNECs is increased by IFN-γ, Th1 cytokines, and decreased by Th2 cytokines, IL-4 or IL-13 (78). Not only the TLR expression but also NFκB expression was increased in CRSwNP compared to healthy control (80).

#### The Epithelial Cell-Derived Cytokines Are Induced by TLR Stimulation

In HNECs, TLR activation promotes the production of many cytokines including TSLP, IL-33, and IL-25. These cytokines are known as epithelial-derived cytokines and have been a focus of interest, due to their ability to activate ILC2 and Th2 cells, resulting in type 2 inflammation.

TSLP is a cytokine belonging to the IL-7 cytokine family. Originally, TSLP was proposed as a regulator to promote differentiation into Th2 cells by activating dendritic cells. Recent studies have suggested that TSLP also plays an essential role in the activation of ILC2, resulting in promotion of type 2 inflammation. Many studies reported increased TSLP expression in CRSwNP compared to control and CRSsNP (81–87).

In HNECs, TSLP expression is induced by TLR stimulation with ligands such as P3CSK4 (TLR2/1) (88) and Poly(I:C) (20, 89). Interestingly, the Poly(I:C)-induced TSLP upregulation in HNECs varies with the inflammatory condition of the donor. Golebski et al. reported that TLR3 stimulation promoted TSLP expression much more in HNECs from CRSwNP than in those from healthy donors (20). Moorehead et al. also demonstrated that Poly(I:C)-induced TSLP induction was higher in HNECs from asthma patients than in those from healthy controls (89). These reports indicate that the microenvironment and inflammatory condition alters the responsiveness of HNEC TLRs with changes in TSLP expression.

IL-33 is one of the cytokines classified into the IL-1 family. Similar to other IL-1 family cytokines, IL-33 also activates NFκB signals and promotes many target proteins. Suppression of tumorigenicity 2 (ST2) is a receptor for IL-33 and is expressed in ILC2. IL-33 promotes ILC2 to release IL-4 and IL-13, resulting in an enhancement of type 2 inflammation. In CRSwNP, several reports have demonstrated increased expression of IL-33 compared to control subjects and CRSsNP (81, 83, 85, 90, 91), while others failed to do so (92–97). This discrepancy might be due to the existence of cleaved or splice variants of IL-33 and different methods used to detect them (98). TLR8 (20) and TLR9 (30) agonists have been shown to induce IL-33 expression in HNECs. In HNECs from CRSwNP, *Aspergillus fumigatus* induced IL-33 expression more than in cells from CRSsNP (94), again indicating that the existence of higher levels of inflammation affects the responsiveness of HNEC TLRs resulting in an increased production of various epithelial-derived cytokines.

IL-25 is another cytokine of the IL-17 family which is also known as IL-17E (99). IL-25 activates NFκB and enhances type 2 inflammation by activating ILC2 and Th2 cells, producing IL-4, IL-5, and IL-13 (99). In HNECs, IL-25 levels, as with other cytokines, were significantly induced by Poly(I:C) application (100). Pam3CysSerLys4 (Pam3CSK4), a ligand for TLR2/1, also upregulated IL-25 although LPS did not (100).

Conflicting results have been reported with regard to IL-25 expression in nasal polyps. Increased IL-25 expression in nasal polyps compared to control and CRSsNP was reported in CRS patients from Asian countries such as China, Japan and Korea (81, 83, 85, 91, 92, 101, 102), while no significant upregulation was reported in CRS patients from the United States and Australia (96, 98). As the inflammatory pattern of nasal polyps is reportedly different between the East and West, these conflicting findings on IL-25 expression in nasal polyps may be due to regional differences (98).

Since these epithelial-derived cytokines are upregulated with TLR stimulation in HNECs, this might well-explain the temporary exacerbation of CRSwNP often found after acute viral infection. In addition, considering that these cytokines are regulated by TLR activation and the downstream signaling cascade, the regulators mentioned above might be possible therapeutic targets for CRSwNP, type 2 inflammatory diseases in the nasal cavity and paranasal sinuses.

### Allergic Rhinitis

Allergic rhinitis (AR) is an IgE-mediated allergic inflammatory condition of the nasal mucosa, characterized by clinical symptoms including sneezing, serous rhinorrhea and nasal obstruction (103). AR is not a life-threatening condition by itself but is a great burden for patients and society through impaired quality of life and socioeconomic impact due to its high prevalence.

#### TLR Expression in Nasal Mucosa From Patients With AR

Recent studies showed that cytokines from epithelial cells are deeply involved in the pathogenesis of AR. As in CRSwNP, the altered expression of TLRs has been reported in nasal mucosa from AR patients. Expression of TLR4, TLR5, and TLR9 was decreased in nasal mucosa from AR patients compared to non-atopic donors (104). The expression of TLRs reportedly changed with exposure to pollen extract. Pollen exposure decreased the expression of TLR1 and TLR6 (105) and increased TLR2, 3, 4, and 8 (105, 106). Besides, pollen-seasonal specific-increased TLR3 expression is reported in AR patients (106). Thus, atopic status appears to affect TLR expression in HNECs (104).

#### Altered TLR Responsiveness in Atopic and Allergic Conditions

Atopic or allergic conditions also affect the responsiveness of the nasal epithelium (104). Globinska et al. showed rhinovirus-induced IFN-γ1 upregulation is significantly less in patients with AR than in healthy individuals (107). Brandelius et al. demonstrated that in the case of HNECs from birch-pollen-AR patients, dsRNA stimulation upregulated IFN-β and IFN-γ1 expression only in pollen season, but not outside pollen season (108). In addition to allergic conditions, seasonal pollen exposure might also affect the responsiveness of TLR ligand-derived cytokine production.

IL-33 induction is more significant in HNECs from atopic patients than in those from healthy controls (104). As discussed above, IL-33 promotes type 2 inflammation, which in turn affects the production of IL-33 (109). It is possible that positive feedback loops exist among TLR stimulus, the epithelial-driven cytokines, and type 2 inflammation in nasal mucosa.

TLR stimulation also affects the allergic condition. Recently, Matsumoto et al. demonstrated that pollen-induced IL-10 expression in peripheral blood mononuclear cells (PBMC) is suppressed by Glucopyranosyl A (GLA), a synthetic TLR4 agonist (110). As IL-10 is known as an anti-inflammatory cytokine and downregulates the expression of type 1 cytokines, TLR stimulation might affect the allergic condition by modulating the expression of type 1 or 2 cytokines.

#### TLR Stimulation Is a Possible Adjuvant for Allergen Immunotherapy

The treatment of AR includes medication, surgery, and allergen immunotherapy (AIT). Among them, AIT is the only disease-modifying treatment for AR (111). TLR agonists have been accepted as possible candidates for adjuvant therapy to increase the efficacy and shorten the course of the therapy (111). Some, but not all, TLR agonists might improve the efficacy of AIT through the promotion of IFNγ-producing Th1 cells and IL-10-producing Regulatory T cells (Tregs) with reduced epithelium-derived cytokines (111). However, the adjuvant effect is not consistent among the TLR agonists but is agonist specific. So far, several clinical trials have been reported, mainly using TLR4 agonists, with promising results (112–115). However, few studies compared the group treated with AIT and adjuvant TLR agonists to those with AIT alone. Further studies are necessary to reveal the contribution of the TLR agonists as a potential adjuvant therapy for AR.

Thus, recent evidence has demonstrated that TLRs in the nasal epithelium are associated with the pathogenesis of CRS and AR through the regulation of cytokines expression. However, the clinical implication of aberrant TLR signaling is still unknown and needs to be studied in detail prior to the development of TLR-signal-targeted therapies. Further research will be necessary to reveal how TLRs affect the pathogenesis of CRS and AR.

## Conclusion

This review provides recent evidence on TLR expression and signaling in HNECs, in the context of pathologies of the sinonasal tract. The epithelium, in addition to providing a physical barrier against microbes, is deeply involved in inflammation, particularly in initiating the inflammatory process against invading microorganisms. The regulatory mechanisms of TLR signals in the nasal epithelium are essential, as dysregulation can directly result in vulnerability to pathogens or exaggerate chronic inflammatory conditions. Indeed, the innate immune mechanisms are deeply involved in the pathogenesis of CRS and AR, the main inflammatory diseases of the sinonasal mucosa with a high prevalence ratio worldwide. In addition, modification of TLR signals could be developed further as a possible adjuvant therapy for AIT. So far, the role of TLR signals in HNECs and in the upper airway is less studied than those in the lower airway. However, this does not infer less importance. Rather, TLR signaling in the upper airway might be vital due to its continuous exposure to microbes. Continuous research on this topic can pave the way to elucidating the pathogenesis for these inflammatory conditions with the potential for the development of new therapeutic targets.

## Author Contributions

MS, AH, and SV designed the study. TS, AN, and YN contributed to collection of data and articles. MS, CC, MR, and SV wrote the draft of manuscript. All authors contributed to the article and approved the submitted version.

## Funding

This study was supported by JSPS KAKENHI Grant Numbers 17H06491, 18K16871, and 18KK0444 and Japanese Society of Allergology, Clinical Research Support Program.

## Conflict of Interest

The authors declare that the research was conducted in the absence of any commercial or financial relationships that could be construed as a potential conflict of interest.

## Publisher's Note

All claims expressed in this article are solely those of the authors and do not necessarily represent those of their affiliated organizations, or those of the publisher, the editors and the reviewers. Any product that may be evaluated in this article, or claim that may be made by its manufacturer, is not guaranteed or endorsed by the publisher.
